# The miR-30 MicroRNA Family Targets *smoothened* to Regulate Hedgehog Signalling in Zebrafish Early Muscle Development

**DOI:** 10.1371/journal.pone.0065170

**Published:** 2013-06-05

**Authors:** Ami Ketley, Anne Warren, Emily Holmes, Martin Gering, A. Aziz Aboobaker, J. David Brook

**Affiliations:** Centre for Genetics and Genomics, University of Nottingham, Nottingham, United Kingdom; Institute of Molecular and Cell Biology, Singapore

## Abstract

The importance of microRNAs in development is now widely accepted. However, identifying the specific targets of individual microRNAs and understanding their biological significance remains a major challenge. We have used the zebrafish model system to evaluate the expression and function of microRNAs potentially involved in muscle development and study their interaction with predicted target genes. We altered expression of the miR-30 microRNA family and generated phenotypes that mimicked misregulation of the Hedgehog pathway. Inhibition of the miR-30 family increases activity of the pathway, resulting in elevated *ptc1* expression and increased numbers of superficial slow-muscle fibres. We show that the transmembrane receptor *smoothened* is a target of this microRNA family. Our results indicate that fine coordination of smoothened activity by the miR-30 family allows the correct specification and differentiation of distinct muscle cell types during zebrafish embryonic development.

## Introduction

Gene regulation during vertebrate embryonic development is complex and requires precise regulation and control. MicroRNAs are small ribonucleic acids, 19–25 nucleotides in length, which fulfil key roles in multiple cellular processes including cell fate specification, cell signalling and organogenesis by acting at the post-transcriptional level to down-regulate the translation of target mRNAs. Nucleotides 2–8 of the microRNA represent the seed sequence and are the most crucial for target binding [1]. Complementarity between this region and an mRNA transcript target is required, but secondary structure and accessibility of the mRNA site are also key factors in target recognition [2], [3]. This makes microRNA target identification complex, and despite extensive investigation little is known about the specific targets of many microRNAs.

The Hh signalling pathway is one of the most extensively studied developmental pathways and is a key regulator of early embryonic development conserved from drosophila to humans [4]–[7]. Hedgehog (Hh) is a morphogen which acts to specify cell fate by establishing a graded distribution in the developing embryo. The timing and concentration of Hh exposure is critical for correct tissue specification [8], [9] and the establishment of an Hh concentration gradient across surrounding cells results in distinct differentiation responses. Multiple developmental systems are affected following disruption of the Hedgehog pathway, including the brain [10] muscle [11]–[14], gastrointestinal system [15] and the limbs [16]–[18] The pathway has also been shown to be critical in the development of numerous cancers, in particular basal cell carcinoma [19].

A number of studies have looked at the potential for microRNA regulation of the Hedgehog (Hh) pathway due to its importance in the induction and patterning of the vertebrate embryo [20] and its strong association with the development of many cancers. MicroRNA dysregulation has been associated with many tumour types and specifically miR-212 has been linked to lung cancer progression via its negative regulatory activity against the Ptc1 receptor [21]. In addition, microarray analysis has determined a subset of microRNAs that demonstrate significant changes in expression as a result of Hh pathway activation levels [22], [23]. The Hh pathway regulator, Suppressor of Fused (SuFu), is directly targeted by miR-214 and this interaction affects muscle fibre specification in the developing zebrafish embryo by regulating the transcription factor *Gli1* and maintaining the required levels of Hh activity in the muscle progenitor cells [20]. A drosophila microRNA cluster, miR-12/miR-283 and miR-304 [24], in addition to miR-960 have been shown to negatively regulate key members of the Hh pathway Smoothened, Costal-2 and Fused [25]. Together this data demonstrates the importance of microRNA regulation in the Hh signalling pathway.

A strong link has been established previously between Hh signalling and the distinct muscle cell types within the developing embryo. Hh signalling is required for the establishment of superficial slow muscle fibres, muscle pioneer cells and a subset of fast muscle fibres; medial fast fibres [26], [27]. Early in development slow muscle progenitor cells are located in the most medial position receiving early Hedgehog signal from the notochord [26]. Lateral cells positioned further from the notochord receive lower levels of Hh signal and differentiate to fast muscle fibres independent of Hh activity. Once specified, slow-muscle cells migrate through the fast muscle precursors to become the most superficial layer of muscle. This movement induces the fast muscle precursors to undergo morphogenesis [13], [27], [28]. Here we report a biological role for the miR-30 family in zebrafish embryonic muscle development by regulation of Hedgehog pathway activity. We observe phenotypic similarities between miR-30 knockdown and Hh misexpression and show that Smoothened protein levels are directly affected *in vivo*. Our results suggest that the miR-30 microRNA family is a critical regulator of muscle cell specification and differentiation.

## Results

### The miR-30 Family is Required for Early Muscle Development

The miR-30 family has been studied extensively and has been used to identify the precise mechanisms of Drosha activity [29], as well as the sequence requirements for miRNA biogenesis and function [30]. The miR-30 family is known to regulate several biological processes, including pancreatic islet cell development [31], mitochondrial fission [32], adipogenesis [33] and osteoblast differentiation [34]. Duisters *et al*. (2009) were the first to report a target, connective tissue growth factor, for miR-30 [35]. Since then, several potential targets of miR-30 regulation have been identified, many of which are implicated in the development of cancer [36]–[38].

**Figure 1:**
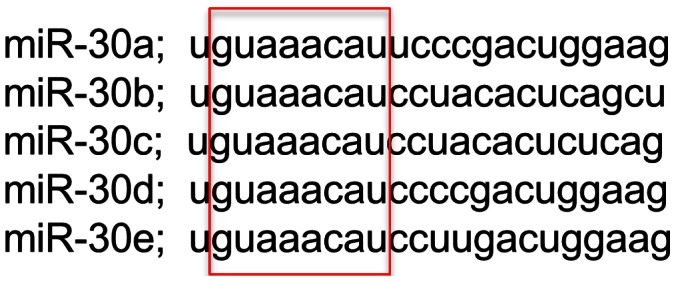
The miR-30 microRNA family shows high sequence similarity and overlapping expression patterns throughout embryonic development. The miR-30 family shows extremely high sequence similarity and an identical seed sequence, as highlighted by the red box.

The family is made up of 5 members, termed miR-30a-30e, between which, the sequence homology is extremely high with 100% conservation in the seed sequence (Fig. 1). The miR-30 family members are encoded from 3 different genomic locations and form 3 microRNA clusters. In order to understand the role of the miR-30 family we conducted a series of experiments using the zebrafish model system. In situ hybridisation with Locked Nucleic Acid (LNA) probes showed that the miR-30 family was detected as early as 8 hpf, unusual for miRNAs in zebrafish [39]. By 26 hpf the expression pattern of miR-30a-30e is overlapping and ubiquitous with noticeable expression in the cerebellum, retina and somites, while miR-30e shows additional expression in the linear heart tube (Fig. S1A). MicroRNA clusters generally demonstrate matching expression profiles, although additional post-transcriptional regulation mechanisms and differing biological contexts are predicted to cause variation in the expression of microRNA genes generated from the same transcripts [40], [41]. Expression analysis of the miR-30 family was carried out in parallel with control experiments using a sense LNA probe for miR-159, as recommended by the manufacturer, which had no detectable expression at the same developmental time points (Fig. S1B).

**Figure 2:**
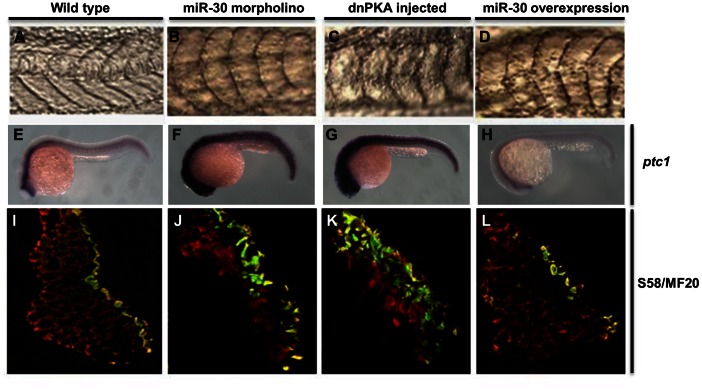
The miR-30 family is required during early embryonic development to regulate Hh pathway activity. Embryo somite structure at 24 hpf is shown (**A–D**). *Ptc1* expression analysis was used as a read out of Hh pathway activity, showing elevated levels in miR-30 morpholino and dnPKA treated embryos when compared to wild type embryos (**E–H**). Slow muscle fibre number was quantified by immunohistochemistry using the S58 antibody (yellow/green) and MF20 staining (red). Embryo sections are orientated dorsal side upwards (**I–L**). Images are shown of wild type embryos (**A, E, I**), miR-30 morpholino treated embryos (**B, F, J**), dnPKA treated embryos (**C, G, K**) and miR-30 overexpression embryos (**D, H, L**).

The miR-30 microRNAs show strong sequence similarity and overlapping expression patterns, which may result in functional redundancy. To assess the role of the entire miR-30 family, a multi-blocking morpholino was designed to knock-down all 5 family members simultaneously in one experiment (Fig. 2). The morpholino was designed to target the pre-mRNA sequence and prevent processing from the primary transcript. The miR-30 family morpholino is 35 bp in length. This spans the entire mature microRNA sequences and the *drosha* and *dicer* cleavage sites. The increased length reduces the percentage of mismatches between family members therefore increasing the probability of complete family knockdown. Morpholino activity was verified using a GFP reporter assay, as described in [20]. A GFP reporter construct was made with the GFP open reading frame followed by perfect target sites for the miR-30 microRNA. This was injected into embryos singly, with the miR-30 RNA and with both the miR-30 RNA and the miR-30 morpholino. This experiment demonstrated the effectiveness of the miR-30 morpholino, as shown by a rescue in the levels of GFP protein. GFP protein was quantified by Western blot and demonstrated 72% inhibition of miR-30 activity by the morpholino (Fig. S2).

MicroRNA-30 family knockdown produced a severe muscle phenotype, (Fig. 2A and 2B) indicating a potentially crucial role in early embryonic development. Previous studies have described minor phenotypic changes as a result of microRNA misexpression, which coincides with the ability of most proteins to tolerate alterations in expression levels [42]. Injection of the miR-30 morpholino yielded embryos with broader, rounded U-shaped somites and alteration of the tail size and structure (Fig. 2B). Embryos displayed a reduction in length of the yolk cell extension, which together with the somite defects resulted in an overall ventral curvature of the embryonic axis.

As a negative control for the knockdown studies an unrelated microRNA was selected to ensure the phenotypes observed were specific to knockdown of the miR-30 family and was not a generic consequence of morpholino introduction. MicroRNA-140 was chosen as it has no reported similarity to any members of the miR-30 family and previous expression analysis in zebrafish has shown that miR-140 is expressed in the palatal skeleton and head cartilage [42], [43] No phenotype was observed in these embryos (Fig. S1C).

### miR-30 Misregulation Affects Hh Pathway Activity

Zebrafish mutants for well characterised molecular pathways have been reported and multiple studies point to the developmental consequences of perturbing Hedgehog (Hh) signalling, which shows acute dosage sensitivity [44]–[46]. We noticed that the phenotype we generated by alterations in the level of the miR-30 family mimics misregulation of the Hh pathway, displaying downwards curvature of the embryos and characteristic U-shaped somites associated with Hh pathway misregulation (Fig. 2B) [14], [47]. To determine whether the miR-30 knock down phenotype was due to a mis-regulation of Hh signalling we analysed *ptc1* expression as a read out of Hh activity (Fig. 2E–H) [48], [49]. *Ptc1* encodes an Hh ligand receptor, transcription of which is activated by Hh signalling [48]. In situ hybridisation of 24 hpf embryos injected with the miR-30 morpholino exhibited increased *ptc1* expression (Fig. 2F) suggesting upregulation of the pathway. As a positive control for Hh pathway activation dnPKA mRNA (dominant negative Protein Kinase A) was injected into zebrafish embryos (Fig. 2C,G,K). Protein kinase A is a negative regulator of Hedgehog signaling and injection of dnPKA leads to overactivation of the pathway [47]. There is significant similarity between the embryos treated with dnPKA and the miR-30 knockdown embryos, with primary defects in the early patterning and establishment of the somites resulting in U shaped somites and overall curvature of the embryo. To further verify that miR-30 levels are linked to Hh pathway activity a miR-30 RNA sequence duplex was overexpressed in zebrafish embryos (Fig. 2D,H,L) and showed reduced *ptc1* expression (Fig. 2H), suggesting that the microRNA family is involved in regulating Hh pathway activity. These experiments indicate that the miR-30 family has a negative regulatory role on the level of Hedgehog signaling during zebrafish embryonic development.

### miR-30 is Required for Correct Specification of the Distinct Muscle Cell Types

Hh signalling is critical to correct muscle specification and studies by others have shown that over-activation of the Hh pathway in the presomitic mesoderm causes a complete switch of presomitic cells to superficial slow-muscle fibre fate at the expense of fast twitch fibres [13], [14], [50]. To evaluate the role of the miR-30 family in muscle development we investigated the effect of miR-30 up- and downregulation on muscle fibre distribution by immunohistochemistry. Antibodies against both slow and fast twitch muscle fibres were used to compare treated embryos and uninjected controls (Fig. 2I–L). Sixty somite sections were analysed for each treatment from 24 hpf embryos. Analysis of the miR-30 morpholino treated embryos showed a significant increase in slow-muscle fibre number and altered distribution to a more internal position within the somite, suggesting an increase in Hedgehog activity (Fig 2J and Table S1). The average slow muscle fibre number in untreated embryo somites was 23.01±3.13 (Fig. 2I), compared to 38.03±9.90 (p<0.0001) in miR-30 morpholino treated embryos (Fig. 2J) and 17.5±6.4 (p<0.0001) in miR-30 overexpression embryos (Fig. 2L). The effect of miR-30 knockdown was compared to the effect of Hh pathway overactivation by injection of dnPKA mRNA [47]. DnPKA treated embryos showed an extremely elevated slow fibre count with an average 55.4±13.90 slow muscle fibres per somite (Fig. 2K).

### miR-30 Acts to Negatively Regulate *Smoothened*

As with most microRNAs, many targets are predicted by algorithms and sequence analysis [51]. Based on such analysis we identified a potential miR-30 target site within the zebrafish 3′UTR sequence of the transmembrane receptor *smoothened (smo)* [52], [53]. Smoothened is a key regulator of Hh pathway activity and is responsible for transducing the signal produced by Shh to the downstream pathway components. In the absence of Hh, Smoothened activity is controlled by Ptc1 inhibition, which is removed following binding of the Hedgehog ligand to the Ptc1 receptor [54]. In situ hybridisation analysis of *smoothened* shows an overlap of expression with miR-30 family members, both temporally and spatially throughout zebrafish embryonic development, allowing for a potential interaction [44].

**Figure 3:**
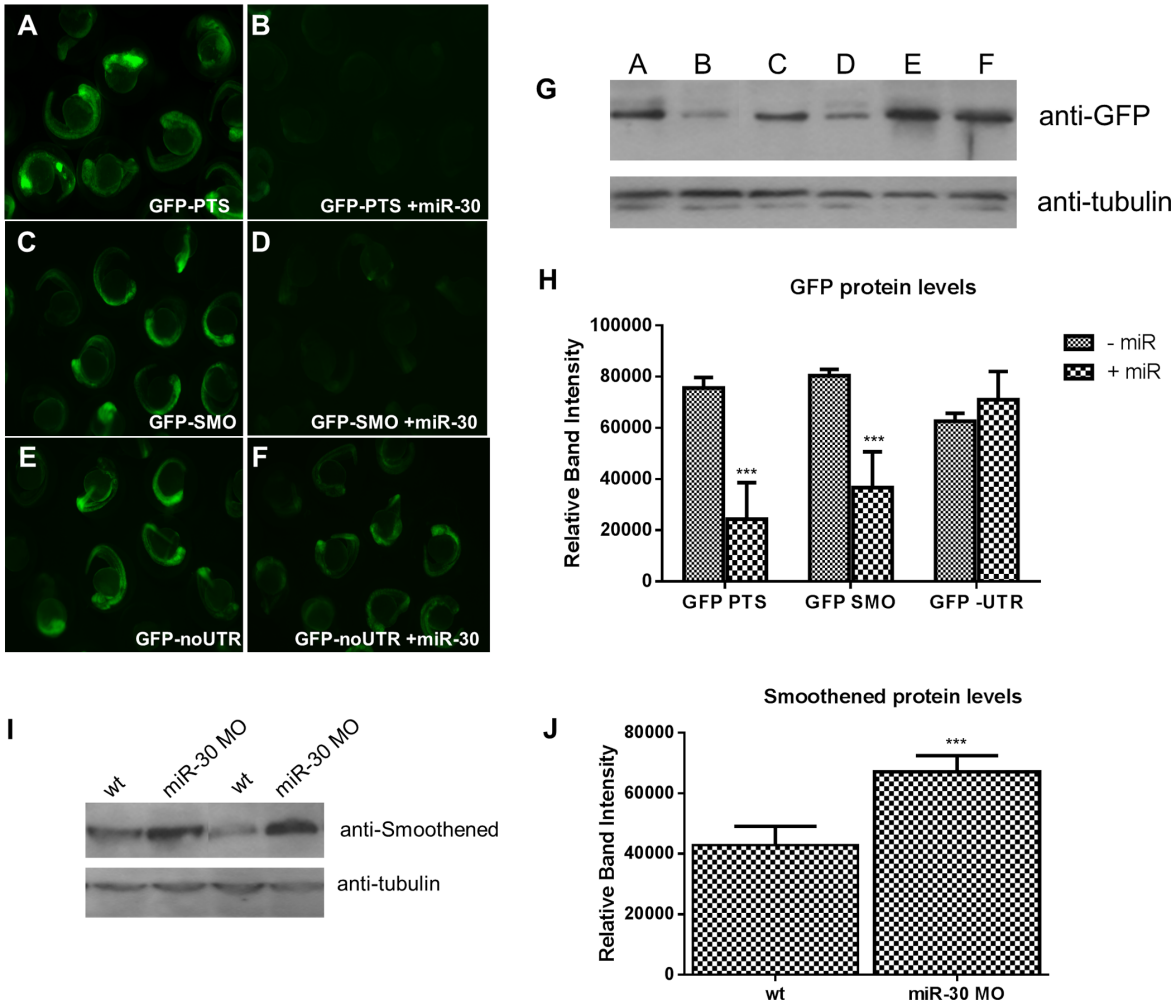
miR-30 directly targets the 3′UTR of the Hedgehog transmembrane receptor smoothened. (**A–F**) Embryos injected with 3 different GFP reporter mRNAs; (**A,B**) the GFP ORF plus tandem perfect target sites (GFP-PTS), (**C,D**) GFP ORF plus the smo 3′UTR sequence (GFP-SMO) and (**E,F**) the GFP ORF without UTR sequence (GFP- no UTR). Constructs were injected either alone (**A,C,E**) or with the miR-30 duplex RNA (**B,D,F**). (**G**) Western blot validation on lysates of GFP injected embryos with and without the miR-30 duplex (**H**) Densitometric analysis of GFP protein levels normalised against α-tubulin loading control shows a 54% reduction in GFP-SMO+miR-30 compared to GFP-SMO only embryos. (**I**) Protein blot analysis of smoothened levels in wild type and miR-30 morpholino knockdown embryos shows an increased level of Smoothened protein. (**J**) Densitometric analysis of the average change in smoothened protein level in 3 samples of wild type versus miR-30 morpholino treated embryos.

To test whether miR-30 directly targets the proposed target site within the *smoothened* 3′UTR, we assessed the ability of miR-30 to negatively regulate three reporter mRNAs. Three different constructs were generated, each containing the GFP ORF followed by either tandem repeats of the miR-30 perfect target site (GFP-PTS) (Fig. 3A–B), an entirely complementary sequence to the microRNA, the *smoothened* 3′UTR sequence (GFP-SMO) (Fig. 3C–D), or no UTR sequence (GFP-no UTR) (Fig. 3E–F) as a negative control. These mRNAs were injected into zebrafish embryos either singly or in combination with the miR-30 duplex sequence. GFP protein expression in embryos was verified using Western Blot analysis on embryo lysates (Fig. 3G). Consistent with a role for the miR-30 family in *smoothened* modulation a 54% reduction was seen in the GFP-SMO+miR-30 embryos (p = 0.0001) (Fig. 3D,G,H) when compared to embryos injected with the GFP mRNAs alone, indicating an interaction between *smoothened* 3′UTR and miR-30. Significantly lower levels of GFP were detected in the GFP-PTS+miR-30 embryos (p<0.0001) (Fig. 3B) and GFP protein levels remained unchanged in embryos injected with GFP- noUTR with or without miR-30 (p = 0.305) (Fig. 3E–F). Further evidence of a direct relationship between miR-30 and *smo* was shown by an increase of 73% in Smoothened protein level following miR-30 morpholino treatment (Fig. 3I–J). This increase was statistically significant with a p value of 0.0069.

**Figure 4:**
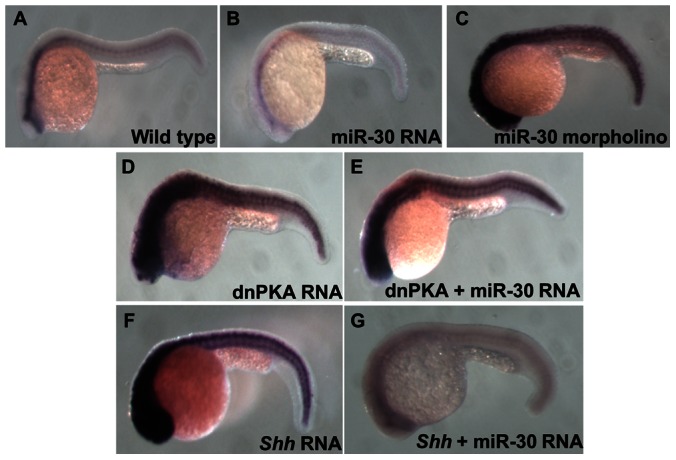
Analysis of Ptc1 reveals the position of miR-30 regulation in the Hh pathway. Ptc1 in situ hybridization shows the level of Hh pathway activity in different embryo treatment types. (**A**) Wild type embryo, (**B**) miR-30 overexpression embryo, (**C**) miR-30 morpholino injected embryo, (**D**) dnPKA overexpression embryo (**E**) dnPKA RNA injected with miR-30 duplex (**F**) *Shh* overexpression embryo (**G**) Reduced *ptc1* expression is seen in *Shh* overexpression embryos coinjected with miR-30 RNA as miR-30 is able to suppress pathway activity.

To establish that Hh pathway activity is regulated by miR-30 via direct targeting of *smoothened*, rather than another pathway component, *ptc1* expression was compared in embryos overexpressing either *Shh* or dnPKA in conjunction with miR-30 (Fig. 4). Sonic hedgehog mRNA was generated from the p64T expression vector, previously described by Krauss et al., 1993 containing the open reading frame of zebrafish *Shh*. The vector was linearised with BamHI and mRNA transcribed with SP6 RNA polymerase, capped and cleaned for microinjection into zebrafish embryos [55]. As shown previously injection of dnPKA RNA leads to an increase in *ptc1* expression (Fig. 4D). Coinjection of dnPKA and miR-30 RNAs also demonstrates elevated *ptc1* levels (Fig. 4E). Consistent with the location of Smoothened upstream of dnPKA in the Hh pathway, overexpression of miR-30 is unable to suppress the effect of dnPKA. However, the overexpression of a more upstream pathway component such as *Shh* (Fig. 4F) is suppressed by miR-30 overexpression (Fig. 4G) indicating the miR-30 target is located between *Shh* and dnPKA in the pathway. The location of the miR-30 target between these two components of the Hh pathway adds further confidence to the hypothesis that *smoothened* is the target gene.

**Figure 5:**
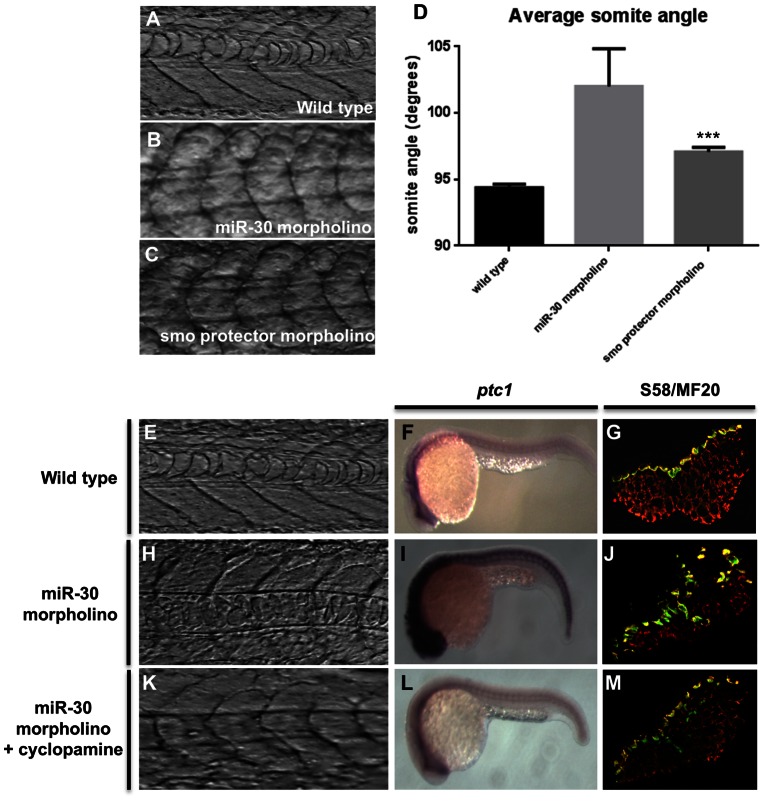
miR-30 acts to negatively regulate smoothened in developing embryos. (**A–D**) Somite angle analysis in wild type, miR-30 morpholino and smoothened protector morpholino injected embryos. Somite structure of (**A**) wild type embryos (**B**) miR-30 morpholino injected embryos (**C**) smoothened protector morpholino injected embryos. (**D**) Histogram to show the average somite angle in wild type and treated embryos. (**E–M**) Cyclopamine treatment causes reversion in somite structure to a more wild type phenotype. Images as shown of wild type embryos (**E,F,G**), miR-30 morpholino embryos (**H,I,J**) and miR-30 morpholino embryos treated with cyclopamine (**K,L,M**). The somite structure of (**E**) wild type (**H**) miR-30 morpholino and (**K**) miR-30 morpholino and cyclopamine treated embryos. (**F, I, L**) Expression of *ptc1* is substantially reduced following cyclopamine treatment (**L**). Embryos are shown with anterior end to the left and dorsal side up. (**G,J,M**) The expanded band of slow fibres, as stained by S58 antibody, is restored to the wild type distribution following cyclopamine treatment (**M**). Embryo sections are orientated dorsal side to the left.

To assess directly the effect of the miR-30-Smoothened interaction on zebrafish muscle structure a smoothened target protector morpholino was injected into embryos and the somite structure analysed at 24 hpf. The protector is complementary to the proposed target sequence within the *smoothened* 3′UTR, and specifically disrupts the miR-30-*smoothened* interaction [56], [57], thus providing valuable information about the physiological role of this pair without the interference of other targets or potential secondary targets [57]. These attributes have been demonstrated in a number of studies of other microRNAs [58]–[61]. Figure 5 shows the somite structure of embryos injected with the target protector. The resulting phenotype was milder than miR-30 family knockdown, however a significant change in somite structure was detected. Angle measurements were taken from wild-type, miR-30 morpholino and protector-injected fish (Fig. 5A–D). All analyses were conducted blind. The mean somite angle in the protector-injected fish (Fig. 5C) was significantly more obtuse than that of the wild-type controls (Fig. 5A) (independent t-test: t = 6.3574, df = 1005, p (one-tailed) <0.0001). The mean angle for wild-type fish was 94.37° (SEM = 0.27), compared to 109.2° (SEM = 2.84) for miR-30 morpholino injected fish and 97.08° (SEM = 0.34) for those injected with the smoothened protector (Fig. 5D).

To confirm that the observed phenotypic, transcript and protein alterations were directly due to miR-30 regulation of *smoothened* we sought to rescue the miR-30 morpholino phenotype using the Smoothened inhibitor cyclopamine (Fig. 5E–M and Fig. S4) [54]. Cyclopamine is a plant derived alkaloid which directly targets Smoothened and consequently inhibits hedgehog signalling [54]. Embryos were injected with the miR-30 morpholino and allowed to develop in water treated with cyclopamine, dissolved in DMSO, at a range of concentrations between 100 µM and 6.25 µM. The optimum cyclopamine concentration for rescue of the miR-30 morpholino phenotype was 6.25 µM, which achieved rescue of the somite structure in 70% of embryos. To evaluate the phenotypic rescue, embryos were monitored up to 24 hpf and the resulting phenotype was assessed for improved overall morphology and somite structure. Cyclopamine rescue yielded miR-30 morpholino treated embryos with more obvious chevron-shaped somites (Fig. 5K). Ventral curvature of the embryos was improved leading to an overall extended morphology similar to that in wild-type embryos (Fig. 5E).

Detailed analysis of the somite structure was carried out on the four somites immediately posterior to the yolk cell extension at 24 hpf following cyclopamine rescue. Analysis of the somite boundaries showed that miR-30 morpholino embryos treated with cyclopamine had an improved angular somite structure (Fig. 5K) that more closely resembled that of the wild type embryo somite (Fig. 5E). In parallel both uninjected and miR-30 morpholino-injected embryos were treated with identical amounts of DMSO to act as a negative control which produced no effect on the phenotypes of the resulting embryos (Fig S4C+D). Furthermore, a reduction in *ptc1* expression was observed following cyclopamine rescue of miR-30 morpholino embryos indicating that Hh pathway activity had been reduced (Fig. 5L). Immunohistochemical analysis revealed that following cyclopamine treatment the number of slow muscle fibres in miR-30 morpholino treated embryos (38.03±9.90) reduced to the wild type range (23.01±3.13) with an average of 24.1±3.58 slow muscle fibres per somite (p = 0.0784) (Fig. 5G, 5J, 5M and Table S1). Together our results indicate that cyclopamine inhibition of Smoothened suppresses the phenotype associated with loss of miR-30 function, supporting the hypothesis that miR-30 modulates Hh signalling by regulation of *smoothened*.

## Discussion

In the current study we have demonstrated that inhibition of the miR-30 microRNA family causes elevated *ptc1* expression and increased numbers of superficial slow muscle fibres during zebrafish muscle development, consistent with an increase in Hh pathway activity. These features are a result of direct targeting of the Hh transmembrane receptor *smoothened* by the microRNA family, representing a novel role for miR-30 in muscle fibre specification and distribution. This is supported by the observation that miR-30 overexpression, and hence Hh pathway activity reduction, can be rescued by coinjection with *Shh* mRNA but not with dnPKA mRNA.

The inhibition of Smoothened is critical to controlled levels of Hh activity within a cell, a function that is attributed to the interaction of the Smoothened protein with Ptc [62]. It has been shown that Ptc acts sub-stoichiometrically to suppress Smoothened, demonstrating a catalytic mode of action rather than a direct interaction between the two pathway components [63]. However, other work has shown that Ptc-mediated inhibition can be overcome by high levels of Smoothened [64]. Here, we show that such an increase in Smoothened protein levels is induced by morpholino-mediated knock-down of the miR-30 family in zebrafish embryos. This increase in Smoothened protein levels leads to an up-regulation of Hh signalling in the developing somites that ultimately results in a very specific muscle fibre patterning defect, namely the development of slow instead of fast muscle fibres. A similar defect had previously been described in embryos in which the Hh pathway had been over-activated by forced expression of Hh ligands or dominant negative PKA in all tissues of the early embryo (35). The phenotype generated from target protection of the miR-30 site within the smoothened mRNA transcript, demonstrating the specific effect of this interaction, produces a defect in early muscle specification resulting in flattened somites and loss of the characteristic chevron structure.

The experiments conducted in this study demonstrate a critical interaction between the miR-30 family and smoothened mRNA in the developing zebrafish embryo. Increased Smoothened levels in the somites results in an abnormal patterning of the muscle fibres. In the miR-30 morphants, Smoothened levels are elevated and as such the somitic cells located more laterally are capable of pathway activation and hence develop into slow rather than fast muscle fibres. In the wild-type embryo only adaxial cells receive a Hh signal strong enough to relieve Ptc-mediated Smoothened inhibition. Our data suggest that in the wild-type embryo miR-30 regulation of *smoothened* mRNA maintains the correct cellular level of Smoothened protein and the appropriate Ptc:Smo ratio to ensure normal patterning of the somitic mesoderm.

Most microRNAs are fine tuning regulators, rather than early developmental switches. In most situations this buffering effect does not have major developmental consequences and microRNAs function to maintain established expression profiles [42]. However, in particular contexts this negative regulatory function has a critical role on key developmental processes [62]. MicroRNA-30 regulation of the Hh pathway, via modulation of Smoothened, represents a prime example of a pathway that is particularly sensitive to changes in its key components in some cell types and therefore microRNA regulation represents an ideal mechanism to maintain the level of control needed for precise activation. By acting to modulate the activity of Smoothened, and subsequently the entire Hh pathway, the miR-30 family undertake a key role in early zebrafish embryonic development.

## Materials and Methods

### In situ Hybridization

Detection of mature microRNAs by in situ hybridization was performed as previously described [39] using digoxigenin (DIG)-labelled Locked Nucleic Acid (LNA) probes (Exiqon). Negative control in situ hybridisation experiments used a sense LNA probe designed against miR-159. Ptc1 in situ hybridisation was conducted following standard techniques. Embryos were pooled and treated for the same hybridisation and staining times.

### Microinjections

Fertilized one-cell zebrafish embryos were injected with 6 ng miR-30 morpholino in 1 nl (TGCATTATTACTCACGGTACGAGTTTGAGTC), 50 pg of miR-30 duplex RNA and 50 pg in vitro-transcribed capped GFP mRNAs. Zebrafish smoothened 3′UTR sequence was amplified by RT-PCR and subcloned downstream of the GFP ORF that was inserted into vector pCS2+. A morpholino designed against smoothened was used to determine antibody specificity, (GAGGACATCTTGGAGACGCAACAAA) and injected at 2.5 ng per embryo (Fig. S3). The smoothened target protector sequence was GTGTATGTAAACACCATAAACTGAC and was injected at 9 ng/embryo.

### Immunohistochemistry

Embryos were immersed in 30% sucrose for 60 minutes and frozen in OCT (R A Lamb) using liquid nitrogen cooled isopentane. 20 µm-thick sections were cut on a cryostat (Microm HM505E) and collected on APES COATED glass slides. Frozen sections were fixed in 1% PFA and blocked in 5% BSA:PBS with triton-X to a final concentration of 0.3%. Antibodies were mouse monoclonal against myosin heavy chain (S58) 1∶50 dilution, and myosin (MF20) 1∶100 dilution. Monoclonal antibodies, S58 developed by F.E. Stockdale and MF20 developed by D.A Fischman, were obtained from the Developmental Studies Hybridoma Bank developed under the auspices of the NICHD and maintained by The University of Iowa, Department of Biology, Iowa City, IA 52242. Secondary antibodies against mouse IgG were Alexafluor labeled 488 (green fluorescent) and 555 (red fluorescent) and used at 1∶300 dilution (Invitrogen). Sections were mounted with Vectashield Mounting Medium with DAPI (Vector).

### Protein Blotting

Blots were probed with antibodies against GFP (Santa Cruz, sc-9996) 1∶200 dilution, α-tubulin (Santa Cruz, sc-5286) 1∶400 dilution, and smoothened (Abcam, ab38686) 1∶1000 dilution. Secondary antibodies were conjugated to HRP and visualized with ECL. Densitometric analysis of protein blots were done using Molecular Dynamics ImageQuant 5.2 software. A commercially available antibody against zebrafish smoothened has not yet been described. However, an antibody raised against part of the human protein, which shares 52% identity with the zebrafish sequence, was predicted to interact with zebrafish smoothened. The specificity of this antibody was tested on a Western blot containing protein from zebrafish in which smoothened levels had been knocked down by morpholino treatment (Fig. S3).

### Cyclopamine Treatment of Zebrafish Embryos

Cyclopamine powder (Toronto Research Chemicals) was dissolved in DMSO. Uninjected and morpholino injected embryos were pooled in group sizes of 30 and exposed to cyclopamine, at 2 hours post fertilisation, at different concentrations diluted in 5 ml of fish water. Cyclopamine concentrations ranged from 100 µM-2.5µM. Control uninjected and injected embryos were treated with identical amounts of DMSO diluted in fish water. Embryos were incubated at 28°C and analysed at 24 hpf.

### Imaging

Brightfield and in situ hybridization embryos were imaged using a Zeiss Lumar V.12 microscope and MTI DC-330 video capture digital camera. Immunohistochemistry treated embryos were imaged using a Zeiss 5.10 confocal microscope. Images were acquired using Improvision Openlab and LSM image software.

### Statistical Analysis

An independent t-test, one-tailed, was used to determine the significance in the somite angle measurements. We performed a two-tailed t-test to determine statistical significance between the number of slow muscle fibres in the different somite sections. Differences were established at a 99% confidence interval.

## Supporting Information

Figure S1 (**A**) Expression of the miR-30 family as determined by in situ hybridisation at 8, 16 and 26 hpf. Embryos are orientated anterior to the left and dorsal up. Expression is ubiquitous with predominant expression in the cerebellum, retina and somites, as indicated (arrows). miR-30e shows additional expression in the linear heart tube (arrowhead) (**B**) Negative control in situ hybridisation using a sense miR-159 LNA probe shows no detectable expression at 8 hpf, 16 hpf and 24 hpf. (**C**) Negative control morpholino against miR-140 showed no detectable phenotype when injected at the same concentration as the miR-30 morpholino upto 3 dpf.
(TIF)

Figure S2 **Validation of the miR-30 morpholino.** (**A**) Injection of zebrafish embryos with GFP fused to a 3′UTR containing (1) tandem miR-30 perfect target sites (GFP-PTS). (2) Co-injection of miR-30 RNA with the GFP-PTS reporter mRNA. (3) Co-injection of miR-30 RNA and the miR-30 morpholino with the GFP-PTS reporter. (**B**) Western blot of embryos as in 1–3 with antibodies against GFP and α-tubulin as a loading control. (**C**) Histogram to quantify the restoration of GFP protein following miR-30 morpholino coinjection. GFP levels are normalised against α-tubulin and presented as a percentage of the GFP-PTS injected embryos.
(TIF)

Figure S3 **Specificity of the human smoothened antibody to the zebrafish smoothened protein.** Specificity of the antibody was tested by Western blot on embryos injected with the miR-wild type embryos and embryos injected with a smoothened morpholino. The substantially reduced band at 32 kDa in the smoothened morpholino treated embryos shows cross reactivity of the human antibody with the zebrafish protein and allowed for quantification of smoothened protein levels.
(TIF)

Figure S4 **Cyclopamine treatment rescues the miR-30 morpholino phenotype.** To achieve phenotypic rescue of the miR-30 morpholino phenotype cyclopamine was used at a concentration range of 100 µM-6.25 µM. At 6.25 µM the miR-30 morpholino phenotype improved to resemble the wild type phenotype with elongation of the tail and improved somite structure (**F**). Cyclopamine was dissolved in DMSO and both wild type and miR-30 morpholino injected embryos were treated with DMSO as a negative control (**A–D**) which had no effect on embryo development when compared to untreated. Wild type embryos treated with 6.25 µM cyclopamine showed a mild phenotype associated with Hh pathway inactivation with U shaped somites and a loss of brain chamber definition (**E**).
(TIF)

Table S1 **Number of muscle cell types in miR-30 morpholino treated embryos.** Slow muscle fibres were visualised by fluorescent immunohistochemistry as in figures 2 and 5. Values are the mean slow muscle fibre number per somite. The number of somites analysed of each embryo type is 60. We performed a two-tailed t-test to establish significance within a 99% confidence interval.
(TIF)
