# Correction: The miR-30 MicroRNA Family Targets *smoothened* to Regulate Hedgehog Signalling in Zebrafish Early Muscle Development

**DOI:** 10.1371/annotation/4e68e9f8-74c0-4c45-828f-7d51786c13e2

**Published:** 2013-11-06

**Authors:** Ami Ketley, Anne Warren, Emily Holmes, Martin Gering, A. Aziz Aboobaker, J. David Brook

There is an incorrect sequence listed in the Microinjections section of the Materials and Methods. The corrected section is:

Fertilized one-cell zebrafish embryos were injected with 6 ng miR-30 morpholino in 1 nl (TGCACCAGCTTCCAGTCAAGGATGTTTACAG), 50 pg of miR-30 duplex RNA and 50 pg in vitro-transcribed capped GFP mRNAs. Zebrafish smoothened 3′UTR sequence was amplified by RT-PCR and subcloned downstream of the GFP ORF that was inserted into vector pCS2+. A morpholino designed against smoothened was used to determine antibody specificity, (GAGGACATCTTGGAGACGCAACAAA) and injected at 2.5 ng per embryo (Fig. S3). The smoothened target protector sequence was GTGTATGTAAACACCATAAACTGAC and was injected at 9 ng/embryo. 

