# Supplementary figures and images for: The miR-30 MicroRNA Family Targets smoothened to Regulate Hedgehog Signalling in Zebrafish Early Muscle Development
Source: PLoS One. 2013 Jun 5;8(6):e65170. doi: 10.1371/journal.pone.0065170 (PMC3673911; doi:10.1371/journal.pone.0065170)

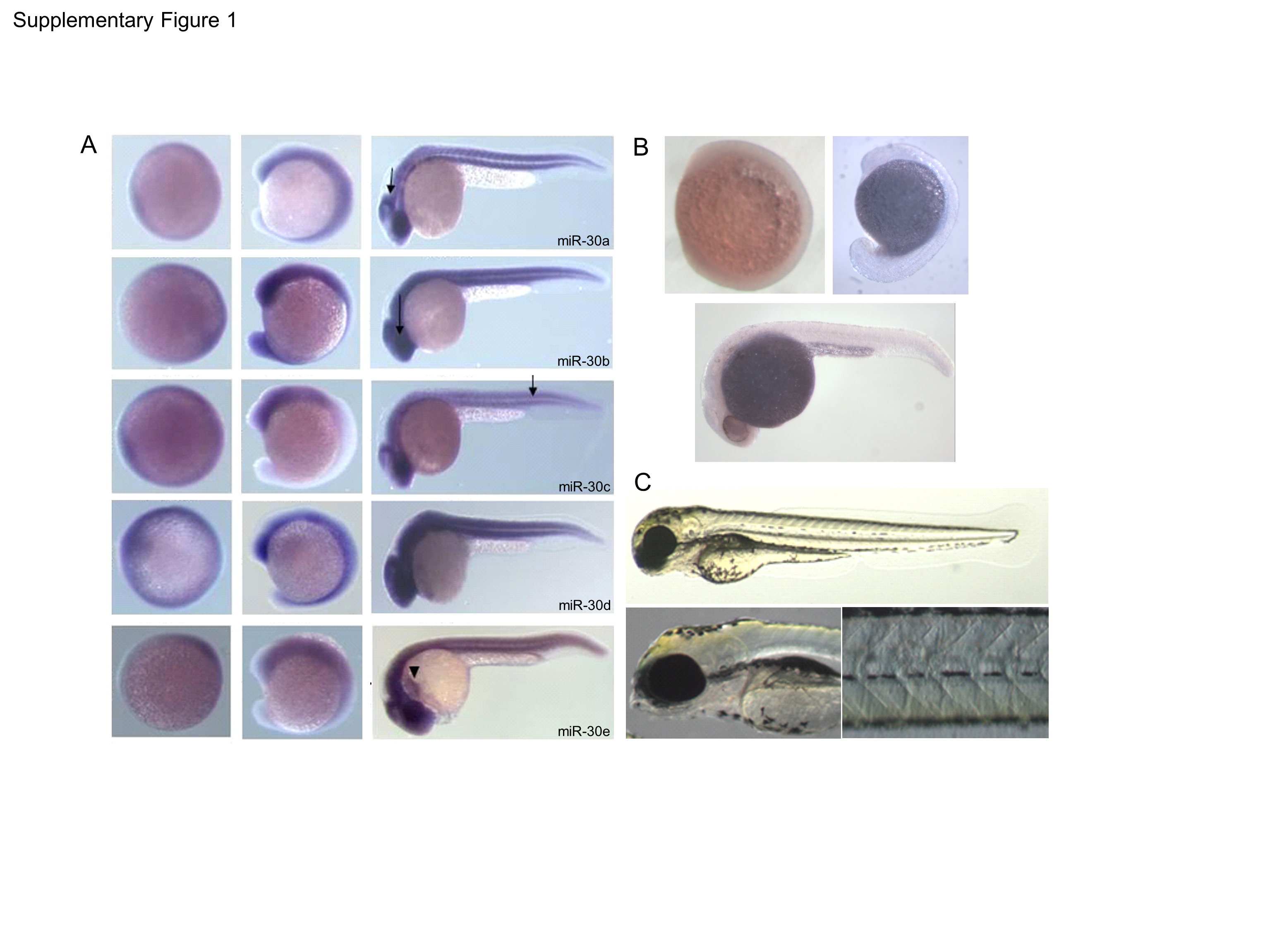

Supplement: Figure S1 — (A) Expression of the miR-30 family as determined by in situ hybridisation at 8, 16 and 26 hpf. Embryos are orientated anterior to the left and dorsal up. Expression is ubiquitous with predominant expression in the cerebellum, retina and somites, as indicated (arrows). miR-30e shows additional expression in the linear heart tube (arrowhead) (B) Negative control in situ hybridisation using a sense miR-159 LNA probe shows no detectable expression at 8 hpf, 16 hpf and 24 hpf. (C) Negative control morpholino against miR-140 showed no detectable phenotype when injected at the same concentration as the miR-30 morpholino upto 3 dpf. (TIF) [file pone.0065170.s001.tif]

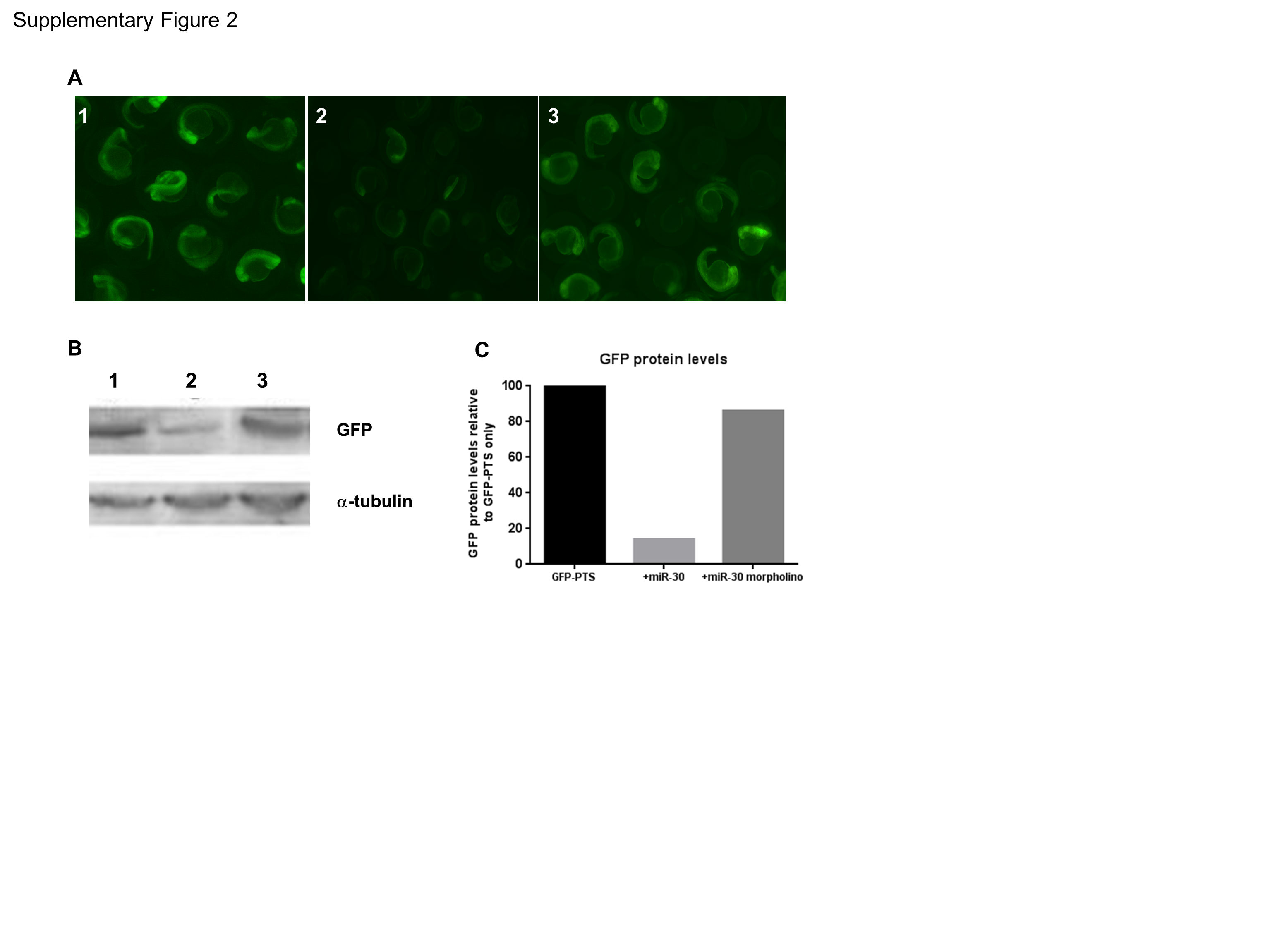

Supplement: Figure S2 — Validation of the miR-30 morpholino. (A) Injection of zebrafish embryos with GFP fused to a 3′UTR containing (1) tandem miR-30 perfect target sites (GFP-PTS). (2) Co-injection of miR-30 RNA with the GFP-PTS reporter mRNA. (3) Co-injection of miR-30 RNA and the miR-30 morpholino with the GFP-PTS reporter. (B) Western blot of embryos as in 1–3 with antibodies against GFP and α-tubulin as a loading control. (C) Histogram to quantify the restoration of GFP protein following miR-30 morpholino coinjection. GFP levels are normalised against α-tubulin and presented as a percentage of the GFP-PTS injected embryos. (TIF) [file pone.0065170.s002.tif]

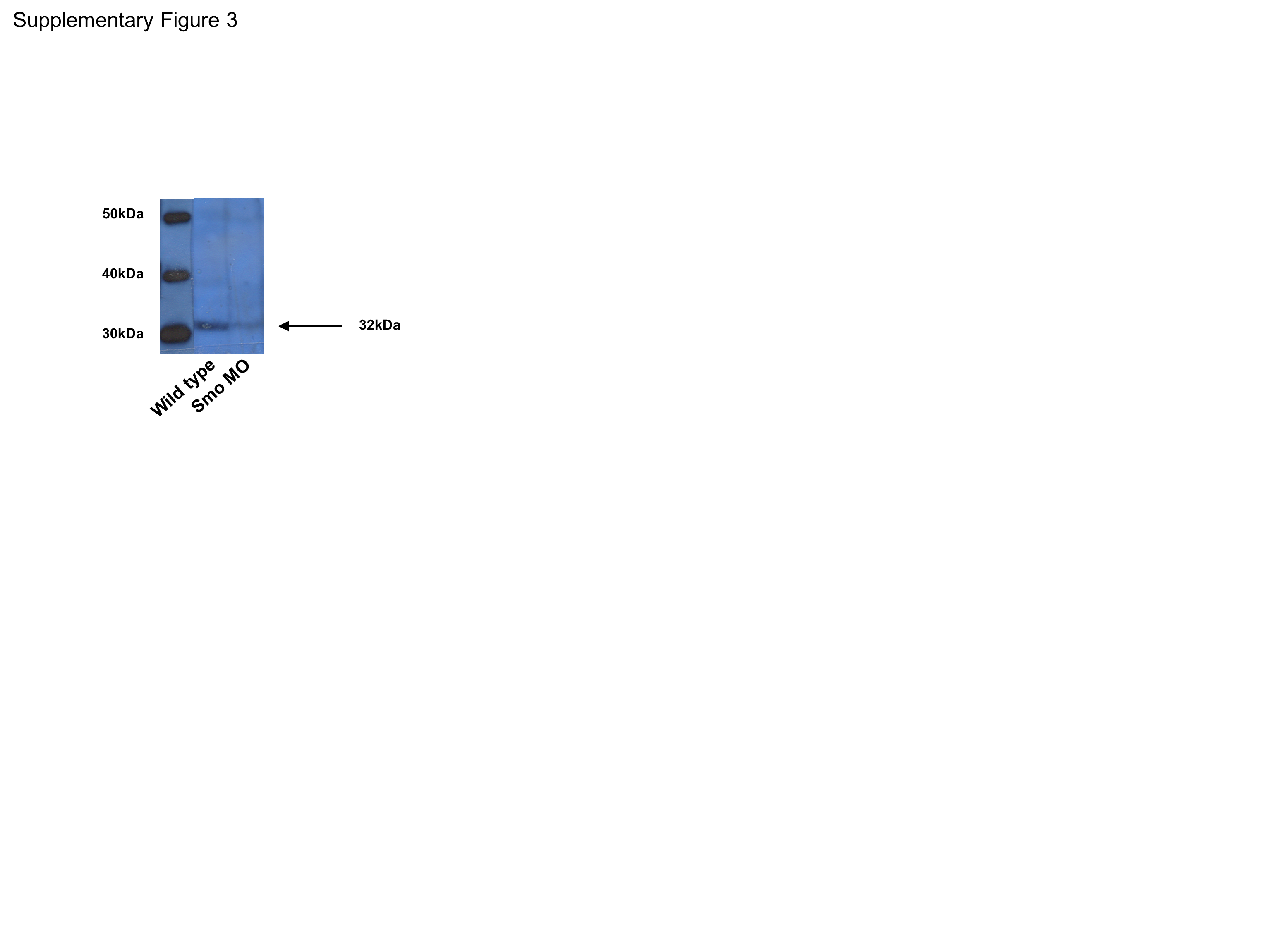

Supplement: Figure S3 — Specificity of the human smoothened antibody to the zebrafish smoothened protein. Specificity of the antibody was tested by Western blot on embryos injected with the miR-wild type embryos and embryos injected with a smoothened morpholino. The substantially reduced band at 32 kDa in the smoothened morpholino treated embryos shows cross reactivity of the human antibody with the zebrafish protein and allowed for quantification of smoothened protein levels. (TIF) [file pone.0065170.s003.tif]

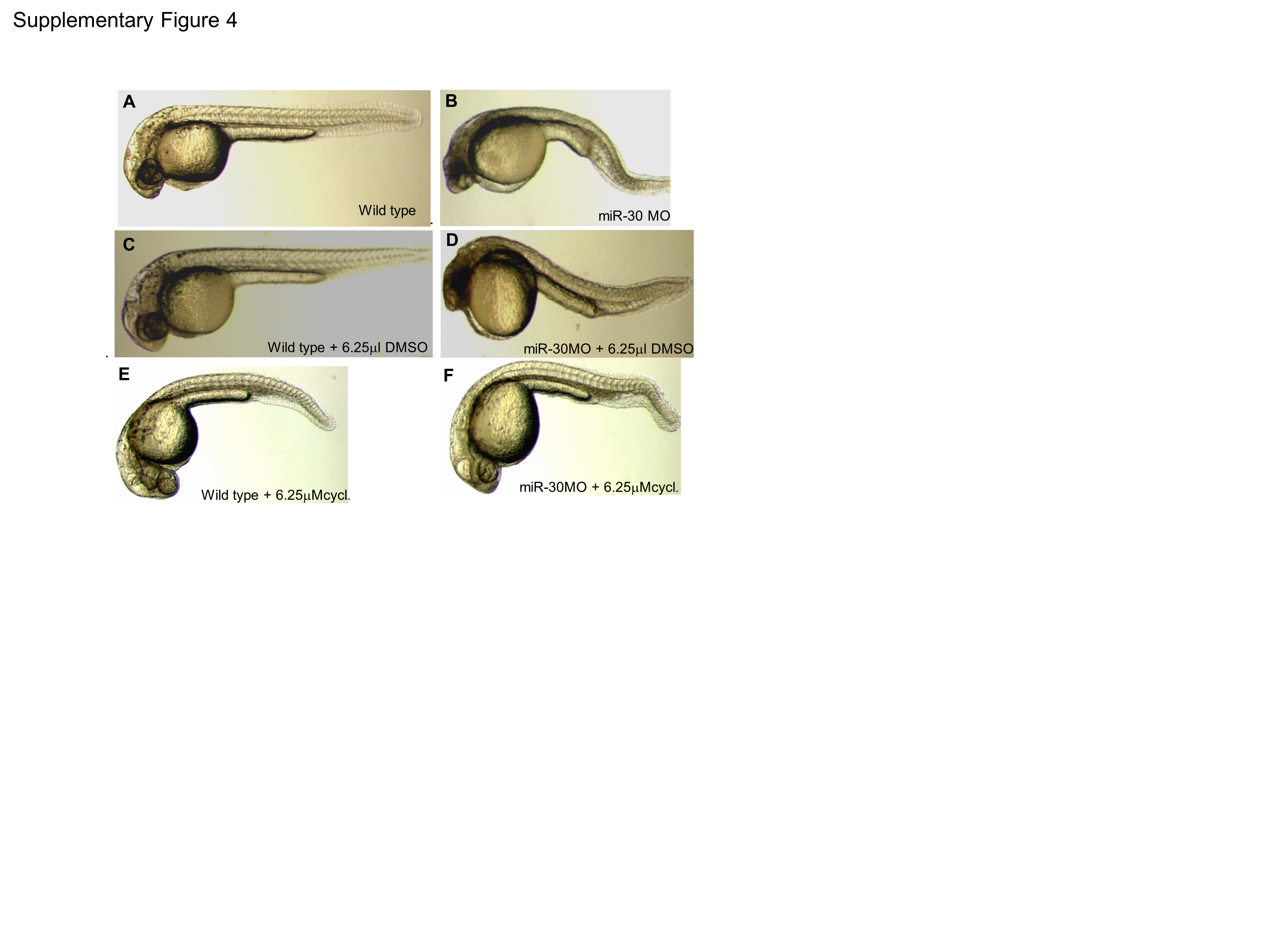

Supplement: Figure S4 — Cyclopamine treatment rescues the miR-30 morpholino phenotype. To achieve phenotypic rescue of the miR-30 morpholino phenotype cyclopamine was used at a concentration range of 100 µM-6.25 µM. At 6.25 µM the miR-30 morpholino phenotype improved to resemble the wild type phenotype with elongation of the tail and improved somite structure (F). Cyclopamine was dissolved in DMSO and both wild type and miR-30 morpholino injected embryos were treated with DMSO as a negative control (A–D) which had no effect on embryo development when compared to untreated. Wild type embryos treated with 6.25 µM cyclopamine showed a mild phenotype associated with Hh pathway inactivation with U shaped somites and a loss of brain chamber definition (E). (TIF) [file pone.0065170.s004.tif]

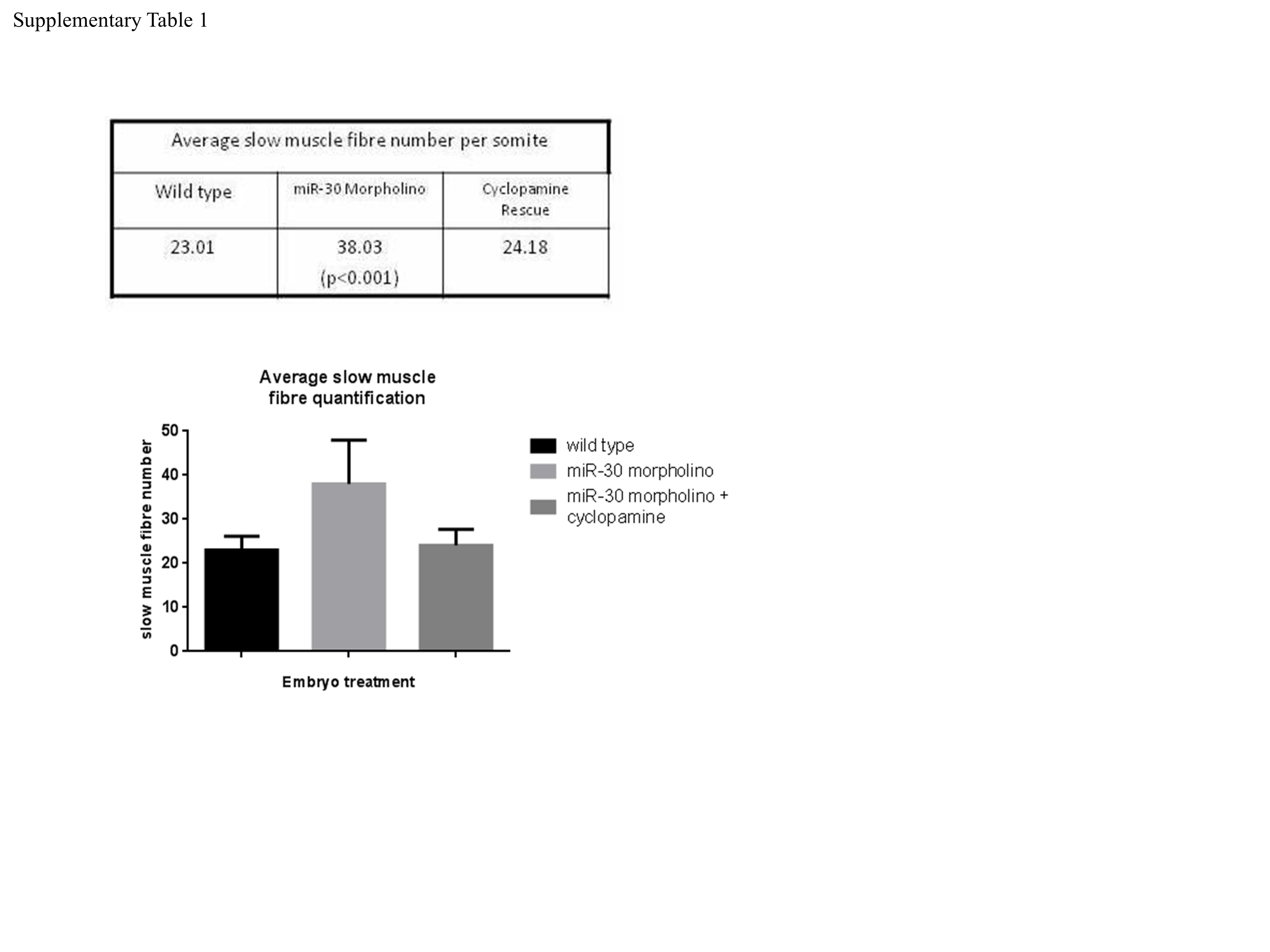

Supplement: Table S1 — Number of muscle cell types in miR-30 morpholino treated embryos. Slow muscle fibres were visualised by fluorescent immunohistochemistry as in figures 2 and 5. Values are the mean slow muscle fibre number per somite. The number of somites analysed of each embryo type is 60. We performed a two-tailed t-test to establish significance within a 99% confidence interval. (TIF) [file pone.0065170.s005.tif]
